# Deciphering the virulent *Vibrio harveyi* causing spoilage in muscle of aquatic crustacean *Litopenaeus vannamei*

**DOI:** 10.1038/s41598-022-20565-1

**Published:** 2022-09-29

**Authors:** Lian Gan, Jianwei Zheng, Wei-Hua Xu, Jianhao Lin, Jingshu Liu, Yu Zhang, Zizhan Wu, Zhaolin Lv, Youming Jia, Qingqi Guo, Shijun Chen, Chuanhe Liu, Tom Defoirdt, Qiwei Qin, Yiying Liu

**Affiliations:** 1 Guangdong-Hong Kong-Macau University Joint Laboratory of Marine Bioresource Conservation and Exploitation, College of Marine Sciences, South China Agricultural University, Guangzhou, China; 2 Guangdong Laboratory for Lingnan Modern Agriculture, Guangzhou, China; 3 Instrumental Analysis and Research Center, South China Agricultural University, Guangzhou, China; 4 Center for Microbial Ecology and Technology (CMET), Ghent University, Ghent, Belgium

**Keywords:** Microbial ecology, Microbial communities, Microbiome, Pathogens, Microbiology, Bacteria, Bacterial host response, Bacterial pathogenesis

## Abstract

The muscle of aquatic crustaceans is perishable and susceptible to environmental contamination. *Vibrio harveyi* is a widely occurring pathogen in aquatic animals. Here, bath treatment with a virulent *V. harveyi* strain (which was added directly in the rearing water to imitate environmental contamination) isolated from the muscle of the whiteleg shrimp, *Litopenaeus vannamei*, caused the muscle of *Li. vannamei* to display a whitish-opaque appearance due to microscopic changes including muscle lysis, muscle fiber damage and microbial colonization. When administered orally by incorporating this isolate in feed (which is an imitation of infection via natural route), rather than direct invasion followed by colonization in the muscle, this isolate indirectly stimulated severe muscle necrosis in *Li. vannamei* via steering the enrichment of two important (human) pathogens, *V. cholerae* and *V. vulnificus*, and one environmental bacterium *Pseudomonas oleovorans*, based on the meta-taxonomic analyses. In addition to the scientifically proven viral diseases, our research proved that bacterial agents are also capable of causing muscle spoilage in crustaceans via changing the microbial composition, and that the crustaceans might be exploited as the wide-spectrum sensitive bio-detector to indicate the extent of microbial contamination.

## Introduction

The ubiquitous aquatic crustaceans are sensitive to versatile biological, physical and chemical pollutants; once affected, they display easy-to-be-recognized disease symptoms, such as a focal to extensive opaque-whitish coloration underneath the carapace. The microbial causative agents of this symptom are conventionally attributed to mainly viruses^1,2^. However, myonecrosis is not inevitably linked to viral infection^3–8^. Recently, a few studies have proven that bacteria belonging to the Vibrionaceae are also capable of causing muscle necrosis in crustaceans; these pathogens include *Vibrio harveyi*, *V. parahaemolyticus*, *Photobacterium damselae* subsp. *damselae*, etc.^3–8^. Meanwhile, with regard to food safety concerns, spoilage of the crustacean muscle is often related to the accumulation of and/or contamination by microbes, including (zoonotic) pathogens, such as *V. cholerae*, *V. vulnificus* and *V. parahaemolyticus*^9^.

*Vibrio* pathogens are ubiquitous in aquatic and terrestrial ecosystems, and able to exert their detrimental effects on a wide range of living organisms, including humans. This study aimed at testing the effects of a virulent *V. harveyi* isolate on the structure and microbiomes of the muscle of *Li. vannamei*, which may provide scientific support for the future construction and application of crustacean-based broad-spectrum bio-detectors to assess microbial contamination in aquatic ecosystems.

## Results and discussion

**Figure 1:**
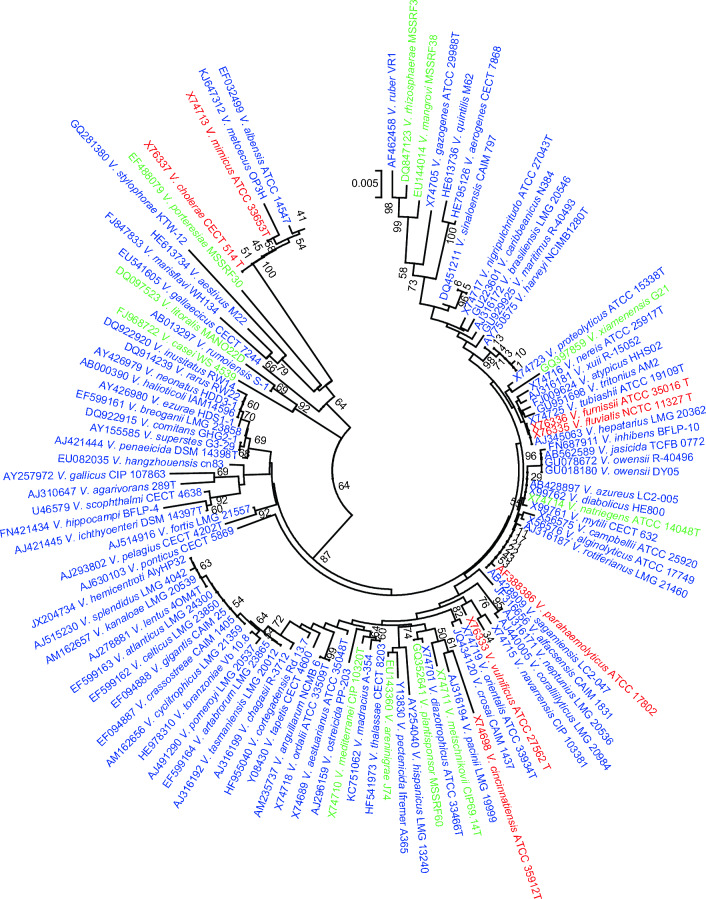
Phylogenetic characterization of *Vibrio* isolates. The neighbor-joining^28^ consensus tree displays 16S rRNA gene sequences (≥ 1200 bp) of (i) 18 *Vibrio* isolates and (ii) all *Vibrio* type strains with good sequence quality (103 strains in total) downloaded from the Ribosomal Database Project^27^ (RDP, http://rdp.cme.msu.edu/). The phylogenetic analyses were performed in Mega 7^29^ using the Kimura-2-parameter^30^ method with Gamma distribution (0.10) to calculate the evolutionary distances. The bootstrap values indicated at the nodes are based on 1 000 bootstrap replicates^31^. Branch values lower than 50% are hidden. The scale bar indicates an evolutionary distance of 0.005 nucleotide substitution per sequence position. Black, red, blue and green colors indicate the isolates of this study and the reference strains from human-related, aquatic and terrestrial/plant sources of isolation, respectively. Strains from (inter)tidal area, coastal sediment, mangrove soil and salt marsh mud are marked in green. The name of each reference strain is preceded by the accession number.

**Figure 2:**
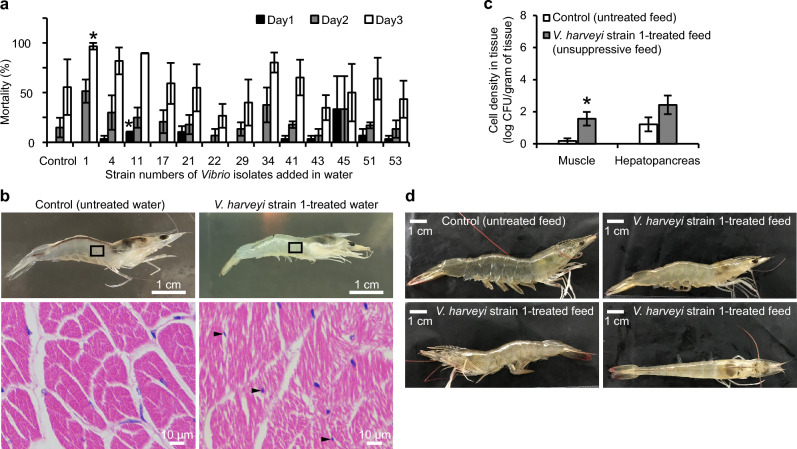
Pathogenicity of *Vibrio* isolates on *Li. vannamei*. (**a**) Mean mortality percentage of *Li. vannamei* after a 3-day challenge with 13 *Vibrio* isolates that proliferate fast in LB broth and (b) the consequential disease symptoms in the muscle of *Li. vannamei* cultivated in *V. harveyi* strain 1-treated water. (**b**) The muscle of the *V. harveyi* strain 1-treated *Li. vannamei* showed extensive whitish-opaque necrosis (upper right), which was not observed in that of the control treatment (upper left). Hematoxylin and eosin (H&E)-stained sections (bottom row) displayed the enlargement of the squared areas of the corresponding *Li. vannamei*. The necrotic muscle (lower right) arranged abnormally with ruptured muscle fibers, degraded muscle and (curved) rod-shaped and bacterial-sized cells (indicated by arrows). (**c**) Bacterial cell density in the muscle and hepatopancreas of *Li. vannamei*, which was determined on TCBS agar after 5 days of feeding with *V. harveyi* strain 1-treated feed in the first feeding test. *Statistically significant difference compared with the corresponding untreated control scored at the same time. Error bars represent S.E.M. (*N* = 3 (**a**) and 24 (**c**)). (**d**) Gross signs of *Li. vannamei* fed with *V. harveyi* strain 1-treated feed, which showed similar symptoms as described in (**b**).

In order to select virulent *Vibrio* isolates, we previously isolated 18 *Vibrio* isolates from the rearing water and the organs (muscle, intestine, hepatopancreas or gill) of *Li. vannamei*^10^. Over 77% of these isolates were taxonomically clustered within the *V. harveyi* and *V. parahaemolyticus* clades (strain 1, 3, 4, 5, 6, 10, 11, 13, 17, 21, 22, 29, 43 and 53), and over 16% within the *V. cholerae* clade (strain 41, 45 and 51) (Fig. 1). To mimic an suboptimal aquatic environment, e.g. pollution by microorganisms and transport of shrimps at high density, a bath challenge test with *Li. vannamei*, focusing on 13 isolates that propagated well in LB broth, revealed that strain 1, which was originally isolated from the muscle of *Li. vannamei*^10^ and showed 99.92% (1288-bp query length of the 16S rRNA gene) homology to *V. harveyi* P4^11^ in the BLASTn comparison on NCBI website and 99.6839% intra-species average nucleotide identity (ANI) to *V. harveyi* FDAARGOS_107^12^ according to the genome-based calculation by FastANI (version 1.32)^13^, exhibited the strongest virulence (Fig. 2a). This isolate was not re-isolated from the muscle of *Li. vannamei* in this test (data not shown), but induced severe whitish-opaque lesions in the muscle (Fig. 2b). Muscle deformation and muscle fiber fracture accompanied by the dispersion of (curved) rod-shaped and bacterial-sized cells were observed photomicrographically (Fig. 2b). Comparing with fishes, shrimps only possess primitive mucosal immunity in their gills^14^, which could not protect them from pathogens of high density^15^. In this test, *Li. vannamei* were immersed in the rearing water with high density of *V. harveyi* strain 1. This pathogen might invade the host via the gills, which were probably the major entrance for the bacteria, and directly go into the hemolymph and ultimately compromise the host.

Unlike most of the vertebrates, crustaceans have microbiota in their hemolymph and the microbiota-immune system balance is pivotal for maintaining their health^16^. In addition, unlike mammals, in which bacteria will be engulfed by macrophages and neutrophils, massive infection in crustaceans will provoke serious melanization; during this process, excessive reactive oxygen species will be secreted into the tissues, which would cause massive tissue damage^17^. According to the genomic analysis of the virulence factors, *V. harveyi* strain 1 harbors genes encoding toxins and toxin secretion, anti-immune strategies to overcome the host immune defenses, strategies to acquire nutritional factors from the host to propagate (which might destroy the microbiota-immunity balance), etc. (Table S1). Collectively, this genomic analysis and the aforementioned experimental results revealed that the pathogenic *V. harveyi* strain 1 might be capable of sabotaging the host immune system, breaking the microbiota-host immune system balance and forcing the host immune system secrete massive reactive oxygen species which cause tissue damage. Therefore, *V. harveyi* strain 1 was used as the infection inoculum in the following feeding tests.

**Figure 3:**
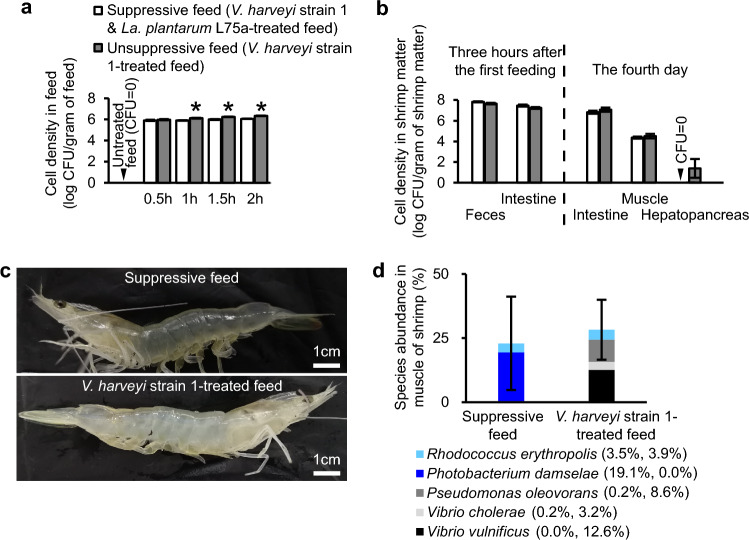
Impact of *V. harveyi* strain 1 on feed pellets and on *Li. vannamei*. The feed pellets were treated with a suspension of *V. harveyi* strain 1 after pre-mixing with (suppressive feed) or without *La. plantarum* L75a-fermented broth. (**a**) Impact of *V. harveyi* strain 1 on the bacterial cell density in feed pellets, which was determined on TCBS agar every half hour in a duration of 2 h. *Statistically significant difference compared with the corresponding suppressive feed treatment scored at the same time. (**b**) Bacterial cell density in the feces and intestine of *Li. vannamei* after 3 h of feeding (left, the second feeding test) and in the intestine, muscle and hepatopancreas after 4 days of feeding (right, the third feeding test), which was determined on TCBS agar. The dotted line separated the results from the two independent tests. (**a**) and (**b**) share the same legends. (**c**) The health status of *Li. vannamei* fed with *V. harveyi* strain 1-treated feed for 4 days (the third feeding test): the diseased *Li. vannamei* (fed without *La. plantarum* supplementation, lower image) exhibited acute whitish-opaque muscle necrosis, while the shrimp fed with the suppressive feed (with *La. plantarum* supplementation, upper image) possessed clear and transparent muscle. These muscle samples were also determined for the percentage of abundance of bacterial species based on the 16S rRNA gene amplicon sequencing (**d**); the detected species without designated species names and/or with mean percentage lower than 3% were not shown; the first and second numbers in the legend represent the percentage of abundance of the corresponding species detected in the muscle of *Li. vannamei* fed with the suppressive feed and *V. harveyi* strain 1-treated diets, respectively. Error bars represent S.E.M. (*N* = 9 (**a**), 8 (**b**) and 3 (**d**)).

In order to investigate the virulence of putative pathogens on aquatic animals, many studies used injection. However, this is not a natural route of infection and thus fails to provide realistic scenarios allowing the tested pathogen to exert authentic pathological activity. Naturally, the vast majority of microorganisms enter their host via feeding. Consequently, the in-taken pathogens can initiate an infection and/or necrosis directly or indirectly (e.g. via stimulating the enrichment or function of other microbes). Indeed, the gastrointestinal tract of *Li. vannamei* has its balanced microbial community composition. The destruction of this balance might negatively affect the immune system and unbalance the hemolymph microbial community in *Li. vannamei*, which could then compromise the shrimps. Therefore, in this research, we studied the potential pathogenicity of *V. harveyi* strain 1 on *Li. vannamei* via inclusion in the diet. Based on the colony counts on the *Vibrio* semi-selective medium TCBS agar (referred to as TCBS-bacteria from this point onwards), in the first feeding test, the cell density of TCBS-bacteria in the muscle of *Li. vannamei* fed with *V. harveyi* strain 1-treated feed was significantly higher compared to that in *Li. vannamei* fed with untreated feed (which contained no detectable TCBS-bacteria as shown in Fig. 3a); while the cell density in the hepatopancreas of the former treatment was higher than that of the later treatment, it showed no significant difference in both treatments (Fig. 2c). Correspondingly, the muscle of the treated *Li. vannamei* showed an extensive whitish-opaque necrotic appearance, while the change in the hepatopancreas was not seen with the unaided eye (Fig. 2d); these might be ascribed to the dissimilar anti-pathogen capability among different organs. Taxonomic analyses of the TCBS-bacteria growing out from these two organs of the treated *Li. vannamei* indicated that they were distantly related to *V. harveyi* strain 1 (Fig. S1). Moreover, a preliminary test revealed that the TCBS-bacteria were not present in the hemolymph collected from the *Li. vannamei* fed with *V. harveyi* strain 1-treated feed (data not shown). Therefore, rather than direct invasion and infection, the ingested *V. harveyi* strain 1 might cause disease in the host via an indirect path, even though this strain was originally isolated from the muscle of *Li. vannamei*.

In further experiments, the whole of the fermented broth of *Lactiplantibacillus plantarum* L75a, which had been preliminarily proven to be antagonistic to several *Vibrio* isolates (including *V. harveyi* strain 1) and not pathogenic to *Li. vannamei*^10^, was introduced as the measure to inhibit *Vibrio* and to guarantee and stabilize the healthy status of *Li. vannamei* in the suppressive treatment (positive control, with *V. harveyi* strain 1 and *La. plantarum* L75a-fermented broth). Under in vitro condition during a 2-h treatment, *V. harveyi* strain 1 facilitated more TCBS-bacteria to grow in the unsuppressive feed pellets (with *V. harveyi* strain 1 and without *La. plantarum* L75a-fermented broth) compared to that in the suppressive feed pellets (Fig. 3a).

Albeit *V. harveyi* is a well-known pathogen associated with *Li. vannamei* and many other aquatic animals, and a few studies have demonstrated that it is capable of causing muscle necrosis in aquatic animals^5,6,18^, the impact of *V. harveyi* on muscle necrosis and the concomitant microbiome composition is hitherto unclear. Parallel to the above in vitro test, we also performed the second and third feeding tests. We found that the orally administered *V. harveyi* strain 1 led to unidentical but not significantly different cell density of TCBS-bacteria in the fecal matter, intestine, muscle and hepatopancreas of *Li. vannamei* fed with both diets (Fig. 3b). Remarkably, the presence of *V. harveyi* strain 1 consistently and conspicuously stimulated acute extensive muscle necrosis in *Li. vannamei* fed with the *V. harveyi* strain 1-treated feed (without *La. plantarum* L75a-fermented broth), which was not observed in *Li. vannamei* fed with the suppressive feed (Fig. 3c).

In addition to being the bio-indicator of microbial contamination, the coloration and appearance of the muscle of shrimps reflect the pathogen composition, which is primarily correlated to food safety^19–21^. Based on the 16S rRNA gene amplicon sequencing of the muscle of *Li. vannamei* from the third feeding test, the abundance of the 4 dominant species detected included *V. cholerae*, *V. vulnificus*, *Pseudomonas oleovorans* and *Ph. damselae*; the former three ones were more abundant in the muscle of the *V. harveyi* strain 1-unsuppressive treatment, while the later one was in reverse (Fig. 3d). With regard to the causative pathogens of zoonotic Vibriosis, *V. cholerae* and *V. vulnificus* are categorized in the ‘higher risk organisms’ whereas *Ph. damselae* and *V. harveyi* in the ‘lower risk organisms’^22^. Notably, *V. cholerae* is affiliated to foodborne diseases in a relatively higher frequency; while *V. vulnificus* and *Ph. damselae* have a higher correlation with contact zoonoses via open wounds, and both of them were sometimes found to be responsible for food spoilage^23,24^. *Ps. oleovorans* was rarely known as a pathogen, and only very few papers reported that it was the causal agent for human infections^25,26^.

In our study, although *V. harveyi* strain 1 was originally isolated from the muscle of *Li. vannamei*, when delivered orally to *Li. vannamei* in the in vivo feeding tests, it was not re-isolated from or detected in the muscle of this host (Figs. 3d, S1, S2). This may imply that, instead of direct invasion, *V. harveyi* strain 1 conferred harmful activity to *Li. vannamei* via facilitating/driving the foodborne pathogens, especially *V. cholerae* and *V. vulnificus*, to invade or proliferate, which compromised the health status of the host. Besides, in the treatment with suppressive feed, although a high density of *Ph. damselae* was detected in the muscle of *Li. vannamei*, it remained healthy. According to the previous studies, *Ph. damselae* subsp. *damselae* was capable of inducing myonecrosis in *Li. vannamei*^3^. Therefore, further studies need to be conducted to elucidate the mechanism of the tripartite interplay amongst the virulent microorganisms (e.g. *Vibrio* pathogens), the host microbiomes and the aquatic hosts, in order to properly apply crustaceans as the bio-detectors for microbial contamination. Importantly, since viruses are commonly asserted as the causative agents of muscle diseases in crustaceans^1,2^, and this study manifested that *V. harveyi* drove the bacterial pathogens in the shrimp muscle to induced easily-visualized disease symptom (myonecrosis), we thus suggest that the crustaceans might be (extensively) applied as straight-forward bio-detectors to indicate the state of (broad-spectrum) microbial contamination in the aquatic ecosystems, especially in the estuary regions that are prone to eutrophication.

In addition to the perspective for practical application, since the four independent in vivo tests (Figs. 2a–d, 3b–d) collectively asserted that the distinctly visible myonecrosis was not directly attributed to the presence of the originally applied pathogen *V. harveyi* strain 1 in the muscle of *Li. vannamei*, these suggested that the contribution could be the necessary cell density and other possible factors to improve or impair beneficial or prejudicial microbiota for causing myonecrosis. This milestone scientific discovery, which does not closely resemble the conventionally known and prevailingly accepted scientific theory, might help ameliorate the existing theory of etiology and pathology of bacterial infections and might be an important starting point of a new research theme in the scientific domain of etiology and pathology of bacterial infections.

## Methods

### Media preparation

The *Vibrio* semi-selective agar, Thiosulfate-Citrate-Bile salts-Sucrose agar (TCBS agar, Guangdong Huankai Microbial Sci. & Tech. Co., Ltd., Guangzhou, Guangdong, China)^10^, was prepared by filling in a stainless still beaker covered with cling wrap and boiling intermittently to avoid boiling over for 1–2 minutes^10^. The Luria–Bertani agar media (LB5 or LB10 agar, consisted of 0.5% or 1% NaCl, respectively; 1% tryptone (Oxoid Ltd., Hampshire, England); 0.5% yeast extract (Oxoid Ltd., Hants, UK); 1.5% agar (BioFroxx GmbH, Einhausen, Germany)) were applied to cultivate *Vibrio* isolates. The Luria–Bertani broth media (LB5 or LB10 broth) were not supplemented with agar.

### Phylogeny of the *Vibrio* isolates

In order to select virulent *Vibrio* isolates, we previously isolated 18 *Vibrio* isolates from the organs (muscle, intestine, hepatopancreas or gill) of *Li. vannamei* and their rearing water^10^ with the salinity of 5 ppt. These *Vibrio* isolates are preserved in 20%-40% glycerol at − 80 °C. The GenBank accession numbers of 16S rRNA gene sequences of these isolates are MT974072-MT974089^10^. These sequences were subjected to phylogenetic analyses. The reference sequences of all 16S rRNA gene sequences of *Vibrio* type strains and of good quality were obtained from the Ribosomal Database Project^27^ (RDP, http://rdp.cme.msu.edu/index.jsp). A neighbor-joining^28^ consensus tree was performed in MEGA7^29^ based on all the 16S rRNA gene sequences (≥ 1200 bp) of the isolates and reference strains. The evolutionary distances were modelled by the Kimura-2-parameter^30^, the non-uniformity of evolutionary rates amongst sites were determined using discrete Gamma distribution, and 1000 bootstrap replicates^31^ were exploited.

### Pre-cultivation and transfer of ***Li. vannamei*** for in vivo tests^10^

The *Li. vannamei* used for the following in vivo tests were purchased from a commercial farm. Upon arrival in the aquaculture laboratory, the *Li. vannamei* were introduced in a recirculation aquaculture system (RAS) containing 0.5% seawater crystal (Yanzhibao™, Guangzhou, Guangdong, China) and cultivated for at least two weeks before use. The muscle of *Li. vannamei* was sampled and detected for absence of bacterial colony on TCBS agar before experiments. Prior to each in vivo test, the *Li. vannamei* were transferred within one hour in the RAS water in big buckets from the RAS to the testing system, which was located in a room with the ambient temperature of 28 ± 2 °C.

### Pathogenicity of the *Vibrio* isolates on *Li. vannamei* via bath treatment

The pathogenicity of the *Vibrio* isolates was determined by a bath challenge test on *Li. vannamei*. Each testing unit, 500 ml glass bottle (with the diameter of 7.5 cm), contained 400 ml of the RAS water and was continuously aerated by an air stone connected to an air pump. Ten *Li. vannamei* of approximately 4 ± 1 cm in length were gently introduced into each testing unit and allowed to acclimatize overnight. To screen for the most virulent *Vibrio* isolate, all isolates that grew well in LB5 or LB10 broth (13 *Vibrio* isolates in total), were tested for their pathogenicity on *Li. vannamei*. Each isolate was pre-grown in LB5 at 28 °C at 200 r.p.m. for 1–2 days. The bacterial cells of each culture were washed three times by sterile RAS water and centrifugation at 5000×*g* for 5 min at room temperature to remove the culture supernatant. The washed cell pellets were resuspended in sterile RAS water. After determination of the cell density by a spectrophotometer at 600 nm, the cell suspension of each isolate was added into each testing unit to reach a final density of 10^5^ cells ml^−1^^32^. Each *Li. vannamei* was fed once daily with one pellet of shrimp feed (type: No. 0, Guangdong Yuehai feed group, Guangdong, China) with the diameter of approximately 2 mm. The dead *Li. vannamei* was removed immediately when observed. The survival of *Li. vannamei* was determined on day 1, 2 and 3. Each treatment was performed in triplicate. The control treatment was not supplemented with *Vibrio* isolate.

### Histological analyses of the muscle of *Li. vannamei*

At the end of the aforementioned bath treatment, two *Li. vannamei*, untreated (control treatment) and bath-treated by the most virulent isolate, *V. harveyi* strain 1, were collected and surface-disinfected by 75% ethanol for 3 s. The muscle of each *Li. vannamei* was dissected and fixed in Bouin's solution for 5 days at room temperature. Then, each muscle sample was processed for examination of hematoxylin and eosin (H&E)-stained paraffin sections by light microscopy. Preparation and photomicrography of the sections were conducted by Wuhan Servicebio Technology Co., Ltd (Wuhan, Hubei, China) according to Servicebio’s in-house protocols.

### The virulence factors of *V. harveyi* strain 1

The two contigs of the genome of *V. harveyi* strain 1 were respectively analyzed by blastn in the database ‘DNA sequences from VFDB full dataset’ on the virulence factor database (VFDB)^33^ website (http://www.mgc.ac.cn/VFs/). From the top 100 sequences producing significant alignments, sequences with identity lower than 80% or related to flagellar structure and function were excluded.

### Pathogenicity of *V. harveyi* strain 1 on *Li. vannamei* via feeding (the first feeding test)

The *Li. vannamei* used for this test was not fed 12 h prior to the test. To circumvent light and perturbation without blocking aeration, each optimized testing unit (plastic box of 37 × 24 × 14 cm) was wrapped with black plastic and covered with a black lid. Each unit contained 8 l of chlorine dioxide-disinfected and dechlorinated RAS water, and was continuously aerated as mentioned above. Five *Li. vannamei* of 10 ± 2 cm were gently introduced into each testing unit and allowed to acclimatize for 4–5 h before the first feeding. The *Li. vannamei* was fed with *V. harveyi* strain 1-treated feed once or twice per day and the feed dry weight of each meal was 0.3% to 1% of their body weight.

To prepare *V. harveyi* strain 1-treated feed, the pregrown cells of *V. harveyi* strain 1 (in LB10 broth) were washed once and resuspended in sterile RAS water to a final density of 10^5^ cells ml^−1^ based on the aforementioned methods. The dry feed of one meal of each testing unit was added in a Petri dish (with the diameter of 6 cm) containing 3–4 ml cell suspension of *V. harveyi* strain 1. After incubation for approximately one hour, the feed was then surface-dried by sterile paper and added into each testing unit. The *Li. vannamei* of the control treatment was fed with untreated feed. Each treatment was performed in quadruplicate (one testing unit represented one replicate). To circumvent water deterioration, dead shrimps, feces and uneaten feed were removed by nets.

After 5 days of cultivation, 8 *Li. vannamei* were collected from each treatment, photographed and surface-disinfected by 75% ethanol for 3 s. Under aseptic condition, the shell of each *Li. vannamei* was removed; and the muscle and hepatopancreas were dissected and put into a sterile 1.5-ml tube containing 200 and 400 µl sterile RAS water, respectively, and two sterile glass beads with the diameter of 3 mm. The muscle and hepatopancreas were weighed and homogenized by a Biologix® vortex mixer (Jinan, Shandong, China) at the maximal speed for 4 and 1 min, respectively. Next, to determine the cell density of bacteria in the two organs that could grow on TCBS agar (referred to as TCBS-bacteria from this point onwards), each homogenized sample was serially diluted by sterile RAS water and spot-inoculated (5 µl per droplet) on TCBS agar in triplicate. The TCBS-bacterial cell density was determined after the TCBS agar plates were incubated at 28 °C for 18 h. To investigate if *V. harveyi* strain 1 was present in the organs, single colonies (with a predilection of yellowish colonies whose morphology was identical to that of *V. harveyi* strain 1) of each treatment and each organ were randomly picked, grown in LB5 broth and sent for 16S rRNA gene sequencing using the forward primer to generate the synthesis of the sequences from the 3’ end. Good-quality sequences (≥ 850 bp) of these colonies were subjected to phylogenetic analyses based on the aforementioned methods.

### *Lactiplantibacillus plantarum* L75a

According to the preliminary tests, the fermented broth of *La. plantarum* L75a, isolated from the intestine of wild *Scylla serrata* from the South China Sea, was inhibitory to the proliferation of several *Vibrio* isolates (including *V. harveyi* strain 1) and was not pathogenic to *Li. vannamei*^10^. Therefore, this fermented broth was exploited as the antagonistic biological agent (in the suppressive treatment) against Vibrios in this study.

### Impact of *V. harveyi* strain 1 on TCBS-bacteria in feed pellets (in vitro test)

According to the aforementioned methods, the *V. harveyi* strain 1-treated feed (*V. harveyi*-unsuppressive feed) was prepared by placing 30 feed pellets in a well of a 6-well cell culture cluster (Costar®, Corning, NY, USA) containing 2 ml cell suspension of *V. harveyi* strain 1. To prepare the *V. harveyi*-suppressive feed, the feed pellets were thoroughly pre-mixed with the whole of the *La. plantarum* L75a-fermented broth and distilled water at the ratio of 100/1/33 (w/w/w) before placing into the cell suspension of *V. harveyi* strain 1. Each treatment was conducted in triplicate. Every 30 min in the duration of 2 h, 2 feed pellets of each treatment were collected by the tweezers, surface-dried by a sterile paper, ground and homogenized by a blunt dissecting needle inside a sterile tube containing 50 µl of sterile 1% saline. To determine the TCBS-bacterial cell density in the feed, each feed suspension was serially diluted by sterile 1% saline and spot-inoculated (5 µl per droplet) on TCBS agar in triplicate. These procedures were conducted in a laminar flow hood to maintain axenic manipulation. The cell density of TCBS-bacteria in the feed was determined after the TCBS plates were incubated at 28 °C for 18 h.

### Impact of *V. harveyi* strain 1 on TCBS-bacteria in the feces, intestine, muscle and hepatopancreas of *Li. vannamei* (the second and third feeding tests)

The experimental setup of the two tests below was similar to the aforementioned feeding test with some adjustments. In both tests, the *Li. vannamei* were fed with *V. harveyi* strain 1-treated feed pre-mixed with (suppressive feed) or without (unsuppressive feed) *La. plantarum* L75a-fermented broth. The *V. harveyi* strain 1-treated feed was prepared based on the methods described in the first feeding test.

In the second feeding test, each testing unit (semi-transparent plastic box of 30 × 20 × 15 cm) contained 5 *Li. vannamei* of 11 ± 2 cm in 6 l of disinfected and dechlorinated RAS water. After acclimation for 4–5 h, the *Li. vannamei* were fed with the aforementioned *V. harveyi* strain 1-suppressive or unsuppressive feed whose dry weight was 1% of their body weight. Each treatment was performed in triplicate (one testing unit represented one replicate). After three hours, fecal strings and intestine of *Li. vannamei* from each testing unit were collected in triplicate. Each sample was surface-dried by sterile paper, weighed, homogenized, suspended and serially diluted in sterile RAS water, spot-inoculated on TCBS agar in duplicate or triplicate, and determined for the TCBS-bacterial cell density according to the aforementioned methods of the first feeding test.

The third feeding test was performed for a longer duration to investigate the impact of *V. harveyi* strain 1 on TCBS-bacteria in the intestine, muscle and hepatopancreas of *Li. vannamei*. Each optimized testing unit, which was used in the first feeding test, contained 5 *Li. vannamei* of 11 ± 2 cm in 8 l of disinfected and dechlorinated RAS water. The first feeding was administered after acclimation for 4–5 h. The *Li. vannamei* were fed twice with the aforementioned *V. harveyi* strain 1-suppressive or unsuppressive feed, and the feed dry weight of each meal was 1% of their body weight. Each treatment was performed in triplicate and each replicate encompassed two testing units. After 4 days of cultivation, one *Li. vannamei* was collected from each replicate, and the intestine, muscle and hepatopancreas were collected and determined for their TCBS-bacterial cell density according to the aforementioned methods of the first feeding test. To investigate if *V. harveyi* strain 1 was present in the muscle of *Li. vannamei* fed with the unsuppressive feed (without *La. plantarum* supplementation), single colonies (with a predilection of yellowish colonies whose morphology was identical to that of *V. harveyi* strain 1) grown from such muscle type were randomly picked, grown in LB10 broth and sent for 16S rRNA gene sequencing. The resultant good-quality sequences, together with the *Vibrio* sequences detected by the 16S rRNA gene amplicon sequencing, were analyzed for their phylogeny based on the aforementioned methods.

### Detection of bacterial community in the muscle of *Li. vannamei *fed with *V. harveyi* strain 1-treated feed

The muscle samples collected in the third feeding test were also sent for 16S rRNA gene amplicon sequencing. DNA extraction, PCR amplification of 16S rRNA gene (with an inclusion of 0.2 µl of BSA within each 20-µl mixture for the PCR reaction), Illumina MiSeq sequencing and processing of sequencing data were performed by Majorbio Bio-Pharm Technology Co. Ltd. (Shanghai, China) according to the protocol described by Wang and colleagues^34^. Briefly, the primers 338F (ACTCCTACGGGAGGCAGCAG) and 806R (GGACTACHVGGGTWTCTAAT) were used. The UPARSE (version7.1, http://drive5.com/uparse/) was applied to cluster the operational taxonomic units (OTUs) with a 97% similarity cutoff^34^.

### Statistics and reproducibility

For each test, the resultant data was analyzed for normal distribution and homogeneity of variances. The impacts of the 13 *Vibrio* isolates on *Li. vannamei*, of *V. harveyi* strain 1 on the feed and on the muscle, hepatopancreas, intestine and feces of *Li. vannamei*, were analyzed by comparing the mortality percentage of the treated *Li. vannamei* (Fig. 2a, *P* < 0.05), by comparing the cell density of TCBS-bacteria in the treated feed (Fig. 3a, *P* < 0.05) and in the treated organs and feces of *Li. vannamei* (Fig. 2c, *P* < 0.05; Fig. 3b, *P* > 0.05), and by comparing the percentage of abundance of detected bacterial species in the muscle of *Li. vannamei* (Fig. 3d, *P* > 0.05), versus their corresponding control treatments scored at the same time using Mann–Whitney *U*-test, so as to ease the comparability amongst different tests. All the statistical tests were two-tailed.

## Supplementary Information

Supplementary Information.

## Data Availability

The accession numbers of the genome of *V. harveyi* strain 1 are CP080097 and CP080098 (BioProject PRJNA749085, BioSample SAMN20353411) in GenBank (www.ncbi.nlm.nih.gov).
