# Author Correction: Deciphering the virulent *Vibrio harveyi* causing spoilage in muscle of aquatic crustacean *Litopenaeus vannamei*

**DOI:** 10.1038/s41598-024-71652-4

**Published:** 2024-09-10

**Authors:** Lian Gan, Jianwei Zheng, Wei-Hua Xu, Jianhao Lin, Jingshu Liu, Yu Zhang, Zizhan Wu, Zhaolin Lv, Youming Jia, Qingqi Guo, Shijun Chen, Chuanhe Liu, Tom Defoirdt, Qiwei Qin, Yiying Liu

**Affiliations:** 1 Guangdong-Hong Kong-Macau University Joint Laboratory of Marine Bioresource Conservation and Exploitation, College of Marine Sciences, South China Agricultural University, Guangzhou, China; 2 Guangdong Laboratory for Lingnan Modern Agriculture, Guangzhou, China; 3 Instrumental Analysis and Research Center, South China Agricultural University, Guangzhou, China; 4 Center for Microbial Ecology and Technology (CMET), Ghent University, Ghent, Belgium

Correction to: *Scientific Reports* 10.1038/s41598-022-20565-1, published online 29 September 2022

The original version of this Article contained an error in Affiliation 1, which was incorrectly given as ‘University Joint Laboratory of Guangdong Province, Hong Kong and Macao Region on Marine Bioresource Conservation and Exploitation, College of Marine Sciences, South China Agricultural University, Guangzhou, China’. The correct affiliation is listed below:

Guangdong-Hong Kong-Macau University Joint Laboratory of Marine Bioresource Conservation and Exploitation, College of Marine Sciences, South China Agricultural University, Guangzhou, China.

The original article has been corrected.

