# Supplementary material for: Deciphering the virulent Vibrio harveyi causing spoilage in muscle of aquatic crustacean Litopenaeus vannamei
Source: Sci Rep. 2022 Sep 29;12:16296. doi: 10.1038/s41598-022-20565-1 (PMC9522882; doi:10.1038/s41598-022-20565-1)
Supplement: Supplementary file 1 — Supplementary Information. [file 41598_2022_20565_MOESM1_ESM.pdf]

**Deciphering the virulent *Vibrio harveyi* causing spoilage in muscle of aquatic crustacean *Litopenaeus vannamei***

Lian Gan, Jianwei Zheng, Wei-Hua Xu, Jianhao Lin, Jingshu Liu, Yu Zhang, Zizhan Wu, Zhaolin Lv, Youming Jia, Qingqi Guo, Shijun Chen, Chuanhe Liu, Tom Defoirdt, Qiwei Qin, Yiying Liu

## Supplementary information

**Table S1 The virulence factors of *Vibrio harveyi* strain 1.** The two contigs of the genome of *V. harveyi* strain 1 were respectively analyzed by blastn in the database 'DNA sequences from VFDB full dataset' on the virulence factor database (VFDB)[1] website (http://www.mgc.ac.cn/VFs/). From the top 100 sequences producing significant alignments, sequences with identity lower than 80% or related to flagellar structure and function were excluded.

| Contig | Sequence producing significant alignment | | | | | BLAST output | | |
|---|---|---|---|---|---|---|---|---|
| | Gene ID (*gene name*) | Product | Related virulence factor | Organism | Length (bp) | Score (bits) | E value | Identity (%) |
| **Contig 1**: | VFG043813 (*VIBHAR_RS18025*) | SGNH/GDSL hydrolase family protein | TLH - Exotoxin | *V. harveyi* ATCC BAA-1116 | 1260 | 394 | e-105 | 82 |
| topology: circular; | VFG007692 (*cpsH*) | Putative capsular polysaccharide synthesis family protein | Capsular polysaccharide - Immune modulation | *V. parahaemolyticus* RIMD 2210633 | 834 | 389 | e-104 | 81 |
| length: 2228839 | VFG007035 (*tlh*) | Thermolabile hemolysin TLH | TLH - Exotoxin | *V. parahaemolyticus* RIMD 2210633 | 1257 | 337 | 2e-88 | 82 |
| bp. | VFG007257 (*vctC*) | Iron chelate ABC transporter ATP-binding protein VctC | VctPDGC system - Nutritional/Metabolic factor | *V. parahaemolyticus* RIMD 2210633 | 756 | 333 | 4e-87 | 80 |
| | VFG007667 (*cpsA*) | Undecaprenyl-phosphate glucose phosphotransferase | Capsular polysaccharide - Immune modulation | *V. parahaemolyticus* RIMD 2210633 | 1401 | 252 | 1e-62 | 82 |
| | VFG007268 (*hutA*) | TonB-dependent hemoglobin/transferrin/lactoferrin family receptor | Heme receptor HutA - Nutritional/Metabolic factor | *V. parahaemolyticus* RIMD 2210633 | 2082 | 252 | 1e-62 | 81 |
| | VFG007669 (*cpsA*) | Undecaprenyl-phosphate glucose phosphotransferase | Capsular polysaccharide - Immune modulation | *V. vulnificus* YJ016 | 1401 | 236 | 7e-58 | 82 |
| | VFG007668 (*cpsA*) | Undecaprenyl-phosphate glucose phosphotransferase | Capsular polysaccharide - Immune modulation | *V. vulnificus* CMCP6 | 1401 | 228 | 2e-55 | 81 |

| | | | | | | | | |
|---|---|---|---|---|---|---|---|---|
| | VFG007261 (*vctA*) | TonB-dependent iron chelate receptor VctA | Enterobactin receptor VctA - Nutritional/Metabolic factor | *V. cholerae* O395 | 1980 | 188 | 1e-43 | 83 |
| | VFG007260 (*vctA*) | TonB-dependent iron chelate receptor VctA | Enterobactin receptor VctA - Nutritional/Metabolic factor | *V. cholerae* O1 biovar El Tor str. N16961 | 1979 | 188 | 1e-43 | 83 |
| | VFG007247 (*vctD*) | Iron chelate uptake ABC transporter permease subunit VctD | VctPDGC system - Nutritional/Metabolic factor | *V. parahaemolyticus* RIMD 2210633 | 936 | 163 | 8e-36 | 83 |
| | VFG007629 (*wzc*) | Polysaccharide biosynthesis tyrosine autokinase | Capsular polysaccharide - Immune modulation | *V. vulnificus* CMCP6 | 2184 | 129 | 1e-25 | 82 |
| | VFG007252 (*vctG*) | Iron chelate uptake ABC transporter permease subunit VctG | VctPDGC system - Nutritional/Metabolic factor | *V. parahaemolyticus* RIMD 2210633 | 951 | 119 | 1e-22 | 81 |
| | VFG044179 (*VP_RS23095*) | Hypothetical protein | Vibrioferrin - Nutritional/Metabolic factor | *V. parahaemolyticus* RIMD 2210633 | 1212 | 117 | 4e-22 | 90 |
| | VFG043634 (*VV1_RS15610*) | CpaF family protein | Flp pili - Adherence | *V. vulnificus* CMCP6 | 1266 | 117 | 4e-22 | 85 |
| | VFG007037 (*tlh*) | Thermolabile hemolysin TLH | TLH - Exotoxin | *V. vulnificus* YJ016 | 1254 | 111 | 3e-20 | 80 |
| | VFG007036 (*tlh*) | Thermolabile hemolysin TLH | TLH - Exotoxin | *V. vulnificus* CMCP6 | 1254 | 109 | 1e-19 | 82 |
| | VFG044181 (*VP_RS23105*) | MFS transporter | Vibrioferrin - Nutritional/Metabolic factor | *V. parahaemolyticus* RIMD 2210633 | 1206 | 105 | 2e-18 | 81 |
| | VFG007630 (*wzc*) | Polysaccharide biosynthesis tyrosine autokinase | Capsular polysaccharide - Immune modulation | *V. vulnificus* YJ016 | 2181 | 105 | 2e-18 | 80 |
| | VFG038380 (*vipB*) | Type VI secretion system contractile sheath large subunit TssC/VipB | T6SS - Effector delivery system | *Aeromonas hydrophila* subsp. *hydrophila* ATCC 7966 | 1479 | 105 | 2e-18 | 87 |
| | VFG007622 (*wza*) | Polysaccharide export protein | Capsular polysaccharide – Immune modulation | *V. vulnificus* CMCP6 | 1137 | 103 | 7e-18 | 87 |
| | VFG007250 (*vctG*) | Iron chelate uptake ABC transporter permease subunit VctG | VctPDGC system - Nutritional/Metabolic factor | *V. cholerae* O1 biovar El Tor str. N16961 | 951 | 101 | 3e-17 | 83 |
| | VFG044182 (*VP_RS23110*) | Siderophore biosynthesis protein PvsD | Vibrioferrin - Nutritional/Metabolic factor | *V. parahaemolyticus* RIMD 2210633 | 1830 | 98 | 4e-16 | 87 |
| | VFG006884 (*tcpI*) | Negative regulator of the major pilin TcpA | TCP - Adherence | *V. cholerae* O395 | 1863 | 88 | 4e-13 | 85 |
| | VFG007695 (*cpsI*) | Glycosyltransferase | Capsular polysaccharide - Immune modulation | *V. parahaemolyticus* RIMD 2210633 | 1032 | 84 | 6e-12 | 81 |
| | VFG007688 (*cpsF*) | Glycosyltransferase | Capsular polysaccharide - Immune modulation | *V. parahaemolyticus* RIMD 2210633 | 1053 | 84 | 6e-12 | 87 |
| | VFG007672 (*cpsB*) | Outer membrane beta-barrel protein | Capsular polysaccharide - Immune modulation | *V. parahaemolyticus* RIMD 2210633 | 1206 | 84 | 6e-12 | 82 |
| | VFG044437 (*huvZ*) | HuvZ protein | HuvA - Nutritional/Metabolic factor | *Listonella anguillarum* H775-3 | 531 | 84 | 6e-12 | 81 |
| | VFG007691 (*cpsG*) | O-antigen ligase family protein | Capsular polysaccharide – Immune modulation | *V. parahaemolyticus* RIMD 2210633 | 1233 | 82 | 2e-11 | 88 |
| | VFG007623 (*wza*) | Polysaccharide export protein | Capsular polysaccharide – Immune modulation | *V. vulnificus* YJ016 | 1137 | 82 | 2e-11 | 85 |

| | | | | | | | | |
|---|---|---|---|---|---|---|---|---|
| | VFG007259 (*vctC*) | Iron chelate ABC transporter ATP-binding protein VctC | VctPDGC system - Nutritional/Metabolic factor | *V. vulnificus* YJ016 | 756 | 82 | 2e-11 | 84 |
| | | | | | | | | |
| **Contig 2**: topology: circular; length: 3866818 bp. | VFG038360 (*ati2*) | Type III secretion system effector Ati2, Inositol polyphosphate 5-phosphatase | T3SS secreted effectors - Effector delivery system | *Aeromonas salmonicida* subsp. *salmonicida* A449 | 1488 | 2305 | 0.0 | 94 |
| | VFG007104 (*epsD*) | Type II secretion system secretin GspD | Eps T2SS - Effector delivery system | *V. parahaemolyticus* RIMD 2210633 | 2028 | 1213 | 0.0 | 83 |
| | VFG007098 (*epsE*) | Type II secretion system ATPase GspE | Eps T2SS - Effector delivery system | *V. parahaemolyticus* RIMD 2210633 | 1503 | 965 | 0.0 | 83 |
| | VFG007183 (*VPA0450*) | Type III secretion system effector | T3SS1 secreted effectors - Effector delivery system | *V. parahaemolyticus* RIMD 2210633 | 1494 | 910 | 0.0 | 85 |
| | VFG006964 (*mshE*) | MSHA biogenesis protein MshE | MSHA pili - Adherence | *V. parahaemolyticus* RIMD 2210633 | 1725 | 811 | 0.0 | 80 |
| | VFG006982 (*mshB*) | MSHA pilin protein MshB | MSHA pili - Adherence | *V. parahaemolyticus* RIMD 2210633 | 498 | 698 | 0.0 | 92 |
| | VFG038361 (*ati1*) | Ati2 chaperone | T3SS - Effector delivery system | *Aeromonas salmonicida* subsp. *salmonicida* A449 | 450 | 630 | e-176 | 92 |
| | VFG006922 (*mshH*) | MSHA biogenesis protein MshH | MSHA pili - Adherence | *V. parahaemolyticus* RIMD 2210633 | 2010 | 626 | e-175 | 84 |
| | VFG006970 (*mshG*) | MSHA biogenesis protein MshG | MSHA pili - Adherence | *V. parahaemolyticus* RIMD 2210633 | 1224 | 587 | e-163 | 81 |
| | VFG007158 (*vscN*) | Type III secretion system ATPase VscN | T3SS1 - Effector delivery system | *V. parahaemolyticus* RIMD 2210633 | 1323 | 569 | e-158 | 80 |
| | VFG007092 (*epsF*) | Type II secretion system inner membrane protein GspF | Eps T2SS - Effector delivery system | *V. parahaemolyticus* RIMD 2210633 | 1218 | 521 | e-143 | 80 |
| | VFG007148 (*vcrH*) | Type III secretion system chaperone VcrH | T3SS1 - Effector delivery system | *V. parahaemolyticus* RIMD 2210633 | 489 | 502 | e-137 | 88 |
| | VFG007086 (*epsG*) | Type II secretion system major pseudopilin GspG | Eps T2SS - Effector delivery system | *V. parahaemolyticus* RIMD 2210633 | 444 | 444 | e-120 | 87 |
| | VFG042880 (*VV1_RS07220*) | PilT/PilU family type 4a pilus ATPase | ChiRP - Adherence | *V. vulnificus* CMCP6 | 1107 | 442 | e-119 | 81 |
| | VFG007147 (*vopB*) | Type III secretion system translocator protein VopB | T3SS1 - Effector delivery system | *V. parahaemolyticus* RIMD 2210633 | 1200 | 424 | e-114 | 80 |
| | VFG006904 (*pilB*) | Type IV-A pilus assembly ATPase PilB | ChiRP - Adherence | *V. parahaemolyticus* RIMD 2210633 | 1686 | 422 | e-114 | 80 |
| | VFG007062 (*epsK*) | Type II secretion system minor pseudopilin GspK | Eps T2SS - Effector delivery system | *V. parahaemolyticus* RIMD 2210633 | 1011 | 420 | e-113 | 86 |
| | VFG007152 (*vcrD*) | Type III secretion system C ring protein VcrD | T3SS1 - Effector delivery system | *V. parahaemolyticus* RIMD 2210633 | 2118 | 410 | e-110 | 85 |
| | VFG006988 (*mshA*) | MSHA pilin protein MshA | MSHA pili - Adherence | *V. parahaemolyticus* RIMD 2210633 | 234 | 333 | 7e-87 | 95 |
| | VFG007186 (*vscN2*) | Type III secretion system ATPase VscN2 | T3SS2 - Effector delivery system | *V. parahaemolyticus* RIMD 2210633 | 1263 | 327 | 4e-85 | 80 |

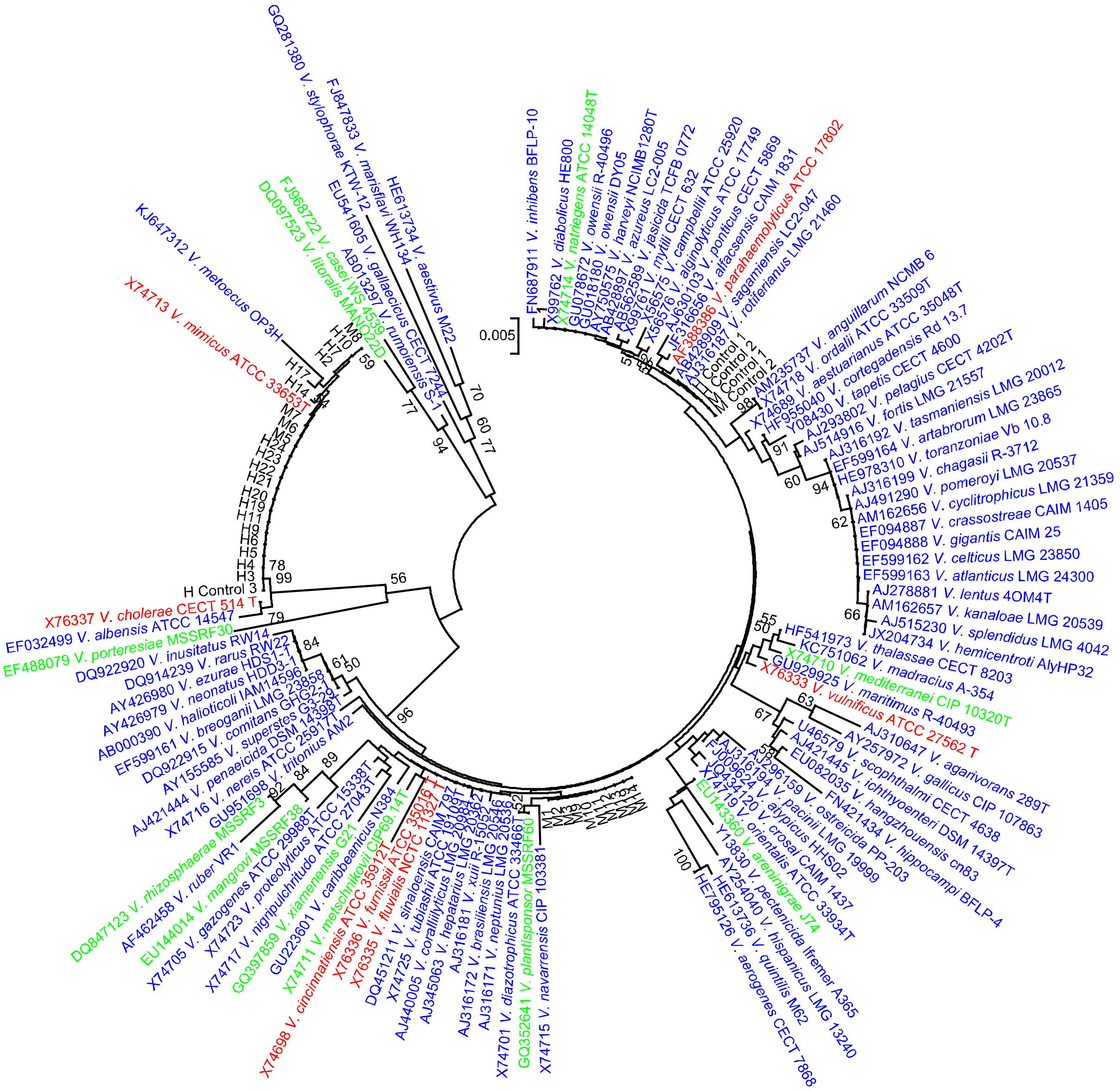


**Fig. S1 Phylogenetic characterization of Vibrios 're-isolated' from the muscle and hepatopancreas of *Litopenaeus vannamei* fed with *V. harveyi* strain 1-treated (unsuppressive) feed in the first feeding test.** The neighbor-joining[2] consensus tree displays 16S rRNA gene sequences (≥ 850 bp) of i) 36 're-isolated' *Vibrio* strains (Figure 2c), ii) *V. harveyi* strain 1 used to treat the feed, and iii) all *Vibrio* type strains with good sequence quality (103 strains in total) downloaded from the Ribosomal Database Project[3] (RDP, http://rdp.cme.msu.edu/). The phylogenetic analyses were performed in Mega 7[4] using the Kimura-2-parameter[5] method with

Gamma distribution (0.11) to calculate the evolutionary distances. The bootstrap values indicated at the nodes are based on 1 000 bootstrap replicates[6]. Branch values lower than 50% are hidden. The scale bar indicates an evolutionary distance of 0.005 nucleotide substitution per sequence position. Red, blue and green colors indicate the reference strains from human-related, aquatic and terrestrial/plant sources of isolation, respectively; black color indicates *V. harveyi* strain 1 and the ‘re-isolated’ strains from the muscle and hepatopancreas (strain numbers preceded by ‘M’ and ‘H’, respectively) of *L. vannamei* in the first feeding test. Strains from (inter)tidal area, coastal sediment, mangrove soil and salt marsh mud are marked in green. The name of each reference strain is preceded by the accession number.

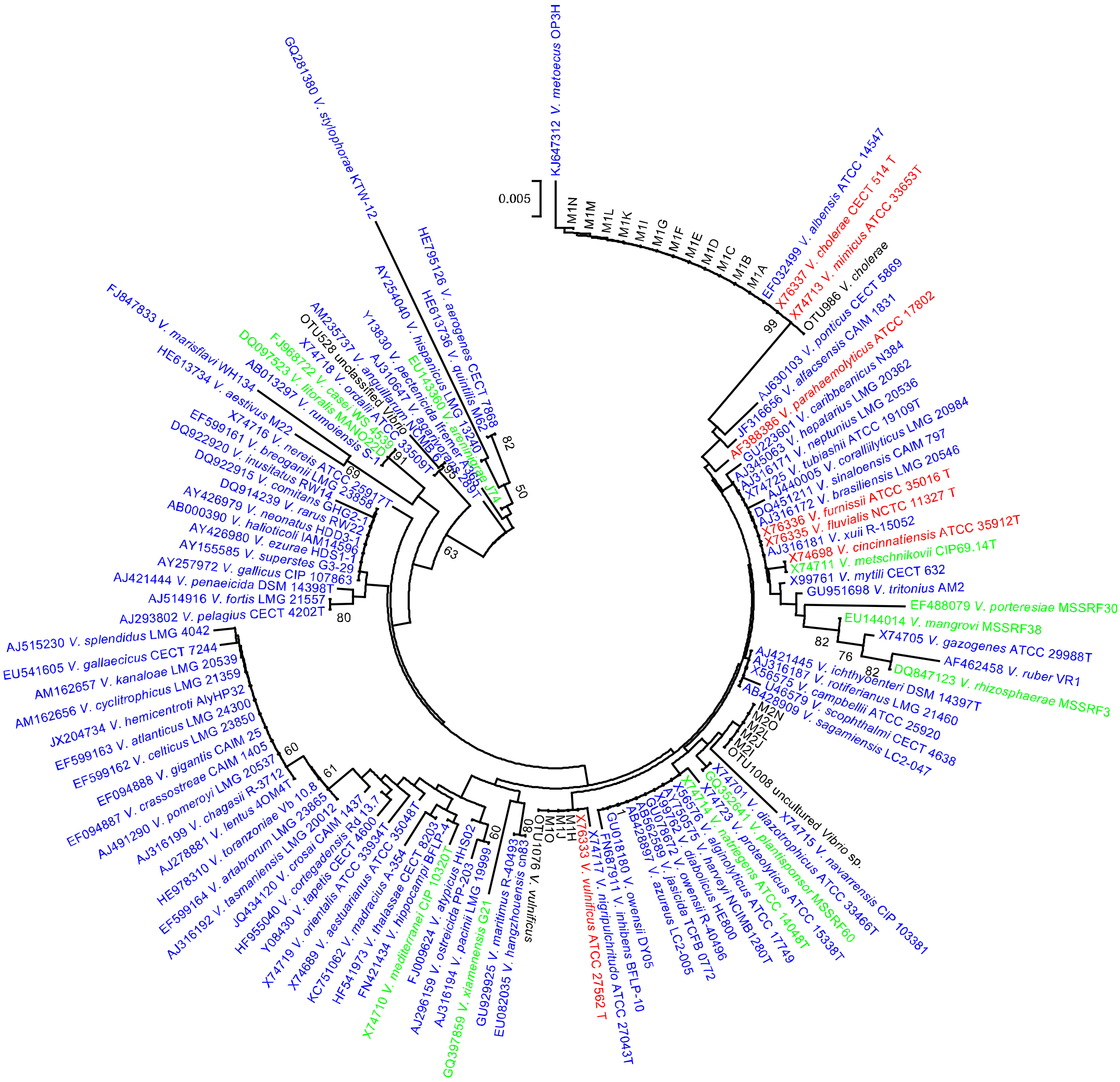


**Fig. S2 Phylogenetically coupling of the 're-isolated' and detected Vibrios from the muscle of *L. vannamei* fed with *V. harveyi* strain 1-treated (unsuppressive) feed in the third feeding test.** The neighbor-joining[2] consensus tree displays 16S rRNA gene sequences (≥ 420 bp) of i) 20 're-isolated' *Vibrio* strains (Figure 3b, right), ii) 4 *Vibrio* OTUs detected by the 16S rRNA gene amplicon sequencing (Figure 3d), iii) *V. harveyi* strain 1 used to treat the feed, and iv) all *Vibrio* type strains with good sequence quality (103 strains in total) downloaded from the Ribosomal Database Project[3] (RDP, http://rdp.cme.msu.edu/). The phylogenetic analyses were performed

in Mega 7[4] using the Kimura-2-parameter[5] method with Gamma distribution (0.10) to calculate the evolutionary distances. The bootstrap values indicated at the nodes are based on 1 000 bootstrap replicates[6]. Branch values lower than 50% are hidden. The scale bar indicates an evolutionary distance of 0.005 nucleotide substitution per sequence position. Red, blue and green colors indicate the reference strains from human-related, aquatic and terrestrial/plant sources of isolation, respectively; black color indicates *V. harveyi* strain 1, the detected OTUs and 're-isolated' strains from the muscle (strain numbers preceded by 'M') of *L. vannamei* fed with *V. harveyi* strain 1-treated (unsuppressive) feed. Strains from (inter)tidal area, coastal sediment, mangrove soil and salt marsh mud are marked in green. M1 and M2 indicate the Vibrios were isolated from the muscle of two *L. vannamei* cultivated in two independent testing units (two biological replicates). The name of each reference strain is preceded by the accession number.

## References


1 Liu, B., Zheng, D., Zhou, S., Chen, L. & Yang, J. VFDB 2022: a general classification scheme for bacterial virulence factors. *Nucleic Acids Research* **50**, D912-D917, doi:10.1093/nar/gkab1107 (2022).

2 Saitou, N. & Nei, M. The neighbor-joining method: a new method for reconstructing phylogenetic trees. *Molecular biology and evolution* **4**, 406-425, doi:10.1093/oxfordjournals.molbev.a040454 (1987).

3 Cole, J. R. *et al.* Ribosomal Database Project: data and tools for high throughput rRNA analysis. *Nucleic Acids Research* **42**, D633-D642, doi:10.1093/nar/gkt1244 (2014).

4 Kumar, S., Stecher, G. & Tamura, K. MEGA7: Molecular Evolutionary Genetics Analysis Version 7.0 for Bigger Datasets. *Molecular Biology and Evolution* **33**, 1870-1874, doi:10.1093/molbev/msw054 (2016).

5 Kimura, M. A simple method for estimating evolutionary rates of base substitutions through comparative studies of nucleotide sequences. *Journal of molecular evolution* **16**, 111-120, doi:10.1007/bf01731581 (1980).

6 Felsenstein, J. Confidence limits on phylogenies: an approach using the bootstrap. *Evolution; international journal of organic evolution* **39**, 783-791, doi:10.1111/j.1558-5646.1985.tb00420.x (1985).
